# Supplementary material for: Gender gap in faculty promotion
Source: PLoS One. 2024 Nov 8;19(11):e0313311. doi: 10.1371/journal.pone.0313311 (PMC11548758; doi:10.1371/journal.pone.0313311)
Supplement: Attachment — Submitted filename: Response to reviewers.pdf [file pone.0313311.s001.pdf]

Response to the comments by the Academic Editor on PONE-D-24-20495

1. *Editor:...a time trend in the gender gap in faculty promotion is not controlled for.*

   In our answer to the reviewer, we now provide results introducing a time trend and its interaction with the gender dummy. The point estimates are reasonable, but the results are largely insignificant due to the lack of statistical power. While we agree that examining the evolution of the gender gap across time is important (as it is likely to decline with time as our point estimate suggests), we do not believe our data allows us to address this question. The average number across time of female researchers that get promoted is 4.6 providing little variation to confidently estimate its evolution across time. We are limited to estimating an average effect. These results are now discussed in the paper at the top of page 9 and in footnote 9.

2. *Editor:...Second, research quality is also time invariant and measured just before entering UNIGE, which is an issue given that the research quality can largely vary over time and differentially by gender.*

   The idea of the research quality variable is not only that it gets rid of the gender bias by taking the ratio of citations to papers as discussed

on page 7 of the paper, but also that by taking this ratio, it captures the intrinsic research quality of the researcher. Indeed, the number of papers and citations will increase as time goes on. The necessary assumption here is that the ratio of these two variables does not vary with time. In our answer to the reviewer we have tried alternative measures and the results are also discussed in footnote 6 of the revised version.

3. *Editor: Third, the empirical strategy does not control for age of the researcher, and therefore, the larger effects found among junior faculty can be related to fertility decisions often occurring at an early stage of the career of a researcher.*

   We agree that fertility decisions can explain part of our results. Unfortunately, we do not have data on the number of children and their date of birth. Controlling for age leads to almost identical results as the ones reported in the paper, and the researcher's age has a point estimate that is virtually 0 and not statistically different from zero. This is probably because it is a distant proxy for fertility decisions. We discuss this avenue for future research in the conclusion of the revised version. Note, however, that there is literature based on qualitative studies suggesting that the explanations for the gender bias in academia do not lie in work-family and fertility issues but rather the hostile environment to women in academia (e.g., Faniko et al. 2022). A quantitative study can help disentangle the importance of these two.

Response to Reviewer #1 on PONE-D-24-20495

We are very grateful for the referee report that raised some important issues and helped us write a much sharper paper. We describe below the changes made to the paper in response to your comments.

1. *Reviewer: The paper uses data from 2004 to 2023. However, the empirical strategy does not control for or exploit a time trend in the gender gap in faculty promotion. This is especially relevant in this context given the gender equal policies implemented in the last few decades in universities. The authors should show whether there is a trend over time in the gender gap in faculty promotion and how this has changed in the 19 years of data used by the authors.*

   This is an important concern as UNIGE policies in terms of how gender bias is addressed have drastically changed during this period. The problem is that the annual intake of assistant and associate professors in a given year is too small to address this question correctly. The average number of entries during the period 2005-2023 is 38.5. Of those 38.5, only 14.2 are female researchers, and only 4.6 female researchers that enter annually get eventually promoted during the period 2004-2023. Trying to get to the trend in promotions and how it differs by gender is difficult with so little statistical power. Nevertheless, we courageously interacted with the gender dummy with a dummy for the year of entry into the sample, and the results are shown in Table A1 below.

Table A1: Introducing time trend in gender discrimination[a]

	(1)	(2)	(3)	(4)
Gender[b]	0.87**	0.51	0.89**	0.57
	(0.06)	(0.36)	(0.05)	(0.62)
PhD[c]			1.45**	1.46
			(0.26)	(0.38)
Quality[d]			1.47***	1.59***
			(0.14)	(0.28)
Time trend (TT)[e]		0.62***		0.68***
		(0.05)		(0.06)
Interaction TT*Gender		1.05		1.04
		(0.09)		(0.10)
School f.e.[f]	No	No	Yes	Yes
LogLikelihood[g]	-277	-231	-251	-214
AIC[h]	560	564	527	456
Observations	674	674	668	668

[a] The first column reproduces the results from the second column in Table 2 of the paper. The second column introduces a time trend and its interaction with the gender dummy to the model in the first column. The third column produces the results of the fourth column in Table 2 of the paper. The fourth column introduces a time trend and its interaction with the gender dummy to the model in the third column. All columns provide the maximum likelihood estimation results for a parametric survival-time Weibull model. All coefficients are reported as hazard ratios. Numbers in parentheses are standard errors of the hazard ratio estimates clustered by school. ⋆ stands for 10 percent statistical significance, ⋆⋆ stands for 5 percent statistical significance, and ⋆⋆⋆ stands for 1 percent statistical significance.

[b] It takes the value 1 for female researchers.

[c] It takes the value 1 for researchers with a PhD from the University of Geneva.

[d] It measures the quality of the researcher when entering UNIGE or in 2004 using the ratio of the total number of citations and the number of published papers.

[e] Time trend (TT) is captured by the year at which the researcher enters the sample.

[f] These are fixed effects for each of the nine schools at the University of Geneva

[g] This is the log pseudo-likelihood.

[h] This is the Akaike Information Criteria, where $AIC = -2\text{LogLikelihood} + 2(c + p + 1)$, with c being the number of covariates, and p is the number of parameters necessary to estimate each model.

As expected neither the gender dummy and its interaction with the time trend are statistically significant in columns (2) and (4). However, the estimated point estimates still signal some important average gender bias of 49 percent when not using school fixed effects or other control variables (column 2) and of 43 percent when using school fixed effects and other control variables (column 4). The interaction of the time trend and the gender dummy is above 1 in both cases, signaling that as time goes on, the gender bias is reduced, as expected by the reviewer. However, the lack of statistical power results in statistically insignificant coefficients. Finally, the time trend suggests that the probability of promotion is lower for individuals who enter the University of Geneva later, but this is just a mechanical effect due to the fact that promotion takes time. We discuss on top of page 9, as well as in footnote 9 in the revised version of the paper.

2. *Reviewer: Data to construct research quality is measured at the time of entry into UNIGE, therefore it is a time-invariant measure. This is a potential limitation given that the research quality can largely vary over time and differentially by gender. Faculty members could enter UNIGE at a very early stage in their career when their research output is still relatively small. For example the six years of tenure track are normally very productive in terms of research output, potentially different by gender and this of course can affect promotion. The authors should control for a measure of research quality measured just before applying for tenure. Alternatively the authors should describe the evolution of research quality by gender over time once joined UNIGE.*

The idea of the research quality variable is not only that it gets rid of the gender bias by taking the ratio of citations to papers as discussed on page 6 of the paper, but also that by taking this ratio, it captures the intrinsic research quality of the researcher. Indeed, the number of papers and citations will increase as time goes on. The necessary assumption here is that the ratio of these two variables does not vary with time. As suggested by the reviewer, it would be great if we could measure research quality at the time of applying for tenure, but unfortunately, we do not have information on the date of application for tenure for those that were denied. We can infer it for those who were granted tenure. What we can do instead is to measure research quality at the time of exit from the sample, which can be due to reaching the year 2023 without a promotion or because the individual quit the University of Geneva for another academic or non-academic job without a promotion. Thus, by construction, this variable will be noisy, and when we introduce it as a control variable, it is still positive and statistically significant, but not to the same extent as when capturing research quality at the time of entry. Table A2 shows the results of these regressions. The first column provides the results of column (4) in Table 2 in the paper, and the second column provides the research quality variable measured at the time of exit (promotion or other reasons).

Table A2: Introducing research quality at the time of exit[a]

	(1)	(2)
Gender[b]	0.89⋆⋆	0.88⋆
	(0.05)	(0.06)
PhD[c]	1.45⋆⋆	1.31⋆
	(0.26)	(0.19)
Quality[d]	1.47⋆⋆⋆	
	(0.14)	
Quality at exit[e]		1.03⋆⋆
		(0.01)
School f.e.[f]	Yes	Yes
LogLikelihood[g]	-251	-264
AIC[h]	527	554
Observations	668	668

[a]The first column reproduces the results of the fourth column in Table 2 of the paper. The second column introduces research quality at the time of exit from the sample. Both columns provide the maximum likelihood estimation results for a parametric survival-time Weibull model. All coefficients are reported as hazard ratios. Numbers in parentheses are standard errors of the hazard ratio estimates clustered by school. ⋆ stands for 10 percent statistical significance, ⋆⋆ stands for 5 percent statistical significance, and ⋆ ⋆ ⋆ stands for 1 percent statistical significance.

[b]It takes the value 1 for female researchers.

[c]It takes the value 1 for researchers with a PhD from the University of Geneva.

[d]It measures the quality of the researcher when entering UNIGE or in 2004 using the ratio of the total number of citations and the number of published papers.

[e]It measures research quality at time of promotion or exit from the sample.

[f]These are fixed effects for each of the nine schools at the University of Geneva

[g]This is the log pseudo-likelihood.

[h]This is the Akaike Information Criteria, where $AIC = -2\text{LogLikelihood} + 2(c + p + 1)$, with c being the number of covariates, and p is the number of parameters necessary to estimate each model.

Results in the second column of Table A2 show that researcher quality when measured at time of promotion or exit is statistically significant but the point estimate is much higher when measured at the time of entry when there is less noise in researchers' motivation. The log likelihood and the AIC statistics also suggest a better fit with the data when measuring researcher quality at the time of entry. Importantly, the estimates on gender bias are unaffected by the use of different measures of researcher quality as shown by the very similar estimates in columns (1) and (2) of Table A2. These results are now also discussed on top of page 7, as well as in footnote 6.

3. *Reviewer: The empirical strategy does not control for the age of the researcher, and therefore, the larger effects found among junior faculty can be related to fertility decisions often occurring at an early stage of the career of a researcher. The authors should control for age of the researcher as well as for fertility decisions. This would be useful to explain some of the channels explaining the gender gap in promotion especially among junior faculty.*

This is a very important point. Unfortunately, we do not have access to data on fertility decisions that would have allowed us to see how they interact with gender bias. Note, however, that one may have to address endogeneity concerns as there may be reverse causality and omitted variable bias (the researcher type may affect both the promotion possibility and fertility decisions as in Sinisalchi and Veronesi,

2020). This important mechanism is now discussed in the conclusion of the paper as an interesting avenue for future research (We are currently exploring with the Ethics Commission at the University of Geneva to have access to such data).

However, we have access to data on the researcher's age at the time of entry into UNIGE. We can, therefore, control for age, which is a distant proxy for fertility. Results are reported in the second column of Table A3. Age does not affect the probability of promotion in a statistically significant way, and other results are qualitatively and quantitatively very similar to those in the first column of Table A3, which reproduces the results of the fourth column in Table 2 in the paper. These results are now discussed in the conclusion.

Table A3: Introducing researchers' age[a]

	(1)	(2)
Gender[b]	0.89⋆⋆	0.89⋆⋆
	(0.05)	(0.05)
PhD[c]	1.45⋆⋆	1.45⋆⋆
	(0.26)	(0.24)
Quality[d]	1.47⋆⋆⋆	1.47⋆⋆⋆
	(0.14)	(0.14)
Age[e]		1.00
		(0.01)
School f.e.[f]	Yes	Yes
LogLikelihood[g]	-251	-251
AIC[h]	527	530
Observations	668	668

[a]The first column reproduces the results of the fourth column in Table 2 of the paper. The second column adds the researcher's age to the same specification. Both columns provide the maximum likelihood estimation results for a parametric survival-time Weibull model. All coefficients are reported as hazard ratios. Numbers in parentheses are standard errors of the hazard ratio estimates clustered by school. ⋆ stands for 10 percent statistical significance, ⋆⋆ stands for 5 percent statistical significance, and ⋆⋆⋆ stands for 1 percent statistical significance.

[b]It takes the value 1 for female researchers.

[c]It takes the value 1 for researchers with a PhD from the University of Geneva.

[d]It measures the quality of the researcher when entering UNIGE or in 2004 using the ratio of the total number of citations and the number of published papers.

[e]It measures research quality at the time of promotion or exit from the sample.

[f]These are fixed effects for each of the nine schools at the University of Geneva

[g]This is the log pseudo-likelihood.

[h]This is the Akaike Information Criteria, where $AIC = -2\text{LogLikelihood} + 2(c + p + 1)$, with c being the number of covariates, and p is the number of parameters necessary to estimate each model.

4. *Reviewer: The empirical strategy takes into account that there might be effects that are school correlated with the gender composition of the school and the promotion across gender. However, these effects that are school specific are very likely to change over time over 19 years of observations. This is another aspect that emphasize that the time effects should be taken into account in the strategy – see point 1 above.*

   We agree with the reviewer. However, as discussed in the answer to the first point, there are not enough observations per cohort to try to address the evolution of gender bias over time with sufficient statistical power. It will be even more difficult to try to address it by school. We acknowledge this shortcoming on top of page 9.

   We also check that results are robust to introducing a variable capturing the year of entry into the University of Geneva. Table A4 reports the results. The first column reports the results of the fourth column in Table 2, and the second column adds the time trend. Apart from the coefficient on the dummy signaling that the researcher has a PhD from the University of Geneva, which becomes statistically insignificant, the results are qualitatively and quantitatively similar.

Table A4: Introducing a time trend[a]

	(1)	(2)
Gender[b]	0.89**	0.80*
	(0.05)	(0.10)
PhD[c]	1.45**	1.45
	(0.26)	(0.40)
Quality[d]	1.47***	1.60***
	(0.14)	(0.26)
Time Trend[e]		0.69***
		(0.05)
School f.e.[f]	Yes	Yes
LogLikelihood[g]	-251	-214
AIC[h]	527	456
Observations	668	668

[a]The first column reproduces the results of the fourth column in Table 2 of the paper. The second column adds a time trend to the same specification. Both columns provide the maximum likelihood estimation results for a parametric survival-time Weibull model. All coefficients are reported as hazard ratios. Numbers in parentheses are standard errors of the hazard ratio estimates clustered by school. $\star$ stands for 10 percent statistical significance, $\star\star$ stands for 5 percent statistical significance, and $\star\star\star$ stands for 1 percent statistical significance.

[b]It takes the value 1 for female researchers.

[c]It takes the value 1 for researchers with a PhD from the University of Geneva.

[d]It measures the quality of the researcher when entering UNIGE or in 2004 using the ratio of the total number of citations and the number of published papers.

[e]It is a variable capturing the year at which the researcher enters UNIGE.

[f]These are fixed effects for each of the nine schools at the University of Geneva

[g]This is the log pseudo-likelihood.

[h]This is the Akaike Information Criteria, where $AIC = -2\text{LogLikelihood} + 2(c + p + 1)$, with c being the number of covariates, and p is the number of parameters necessary to estimate each model.

5. *Reviewer: The paper uses an innovative and convincing way to address the biases in measuring research quality. For this reason I would suggest to emphasize this aspect more in the abstract as well as in the intro by anticipating the analysis provided in support of the unbiasedness of their measure.*

   Thank you for your feedback on the research quality measure. We have now followed your suggestion and have now highlighted the gender unbiasedness of the new measure of research quality in the abstract and the introduction (pages 2 and 3).

6. *Reviewer: Table 1 shows that 38 percent of faculty obtained a promotion in the period 2004-2023. This number seems quite low, considering the 19 years of observations. How does this compare to other universities in Switzerland and other countries?*

   It is 38 percent of the total population of associate and assistant professors at the University of Geneva during the period 2004-2023. It is not the rate of success on promotion applications for which we do not have data, but which it is certainly much higher. Indeed, many researchers leave the university without ever applying or deciding to stay at the University of Geneva but never apply for promotion. We have now clarified this in footnote 7 on page 7.

   Regarding information for other Swiss universities, to our knowledge,

neither the rate of success on promotion applications nor the share of assistant and associate professors who get promoted is publicly available. The best we could find is a document of Swiss universities that reports that during the period 2018-2020, the total number of new hires with respect to the total number of professors in Switzerland was 20 percent. But again, neither the numerator nor denominator in this ratio corresponds to the available data.

7. *Reviewer: To support the findings in Table 3 (end of page 12) the authors should show how the gender gap in hiring has changed over the same period of time.*

As discussed in our answer to point 1 above, the point estimate of the interaction of a time trend with a gender dummy is larger than 1 (see Table A1), suggesting that the gender gap decreases with time over the 2004-2023 period. This is now discussed at the top of page 9.
